# Prognostic value of psoas muscle area and pleural effusion in patients undergoing TAVI

**DOI:** 10.1016/j.ijcha.2026.101871

**Published:** 2026-01-18

**Authors:** Otto Järvinen, Jani Rankinen, Jussi Hernesniemi, Marko Virtanen, Pasi Maaranen, Markku Eskola, Niku Oksala, Juho Tynkkynen

**Affiliations:** a Faculty of Medicine and Health Technology, Tampere University, Tampere, Finland; b Finnish Cardiovascular Research Centre Tampere, Tampere, Finland; c Centre for Vascular Surgery and Interventional Radiology, Tampere University Hospital, Tampere, Finland; d Heart Hospital, Tampere University Hospital, Tampere, Finland

**Keywords:** Sarcopenia, Mortality, Aortic valve stenosis, Frailty, Transcatheter aortic valve replacement

## Abstract

**Background:**

Radiographic markers such as psoas muscle area (PMA) and pleural effusion have been linked to mortality after transcatheter aortic valve implantation (TAVI). We examined their relationship with cause-specific mortality and their incremental prognostic value beyond EuroSCORE II.

**Methods:**

This retrospective study included 1090 consecutive TAVI patients treated at Heart Hospital, Tampere University Hospital between 2008 and 2020. Preoperative CT scans were reviewed for L3-level PMA and pleural effusion (>10 mm thickness). Subdistribution hazard models adjusted for age, sex, BMI, and BSA were used to analyze cause-specific mortality. Incremental prognostic value beyond EuroSCORE II was assessed using time-dependent discrimination indexes (AUC and IDI) and net-reclassification index (NRI) at 3 years.

**Results:**

During a median follow-up of 4.3 years (IQR 3.1–6.0), 54% (n = 590) of patients died: 64% (n = 376) from cardiovascular, 30% (n = 177) from non-cardiovascular, and 6% (n = 37) from unnatural causes. PMA and pleural effusion were associated with cardiovascular mortality (PMA: SDH/1SD 0.88, 95% CI 0.78–0.99, p = 0.037; pleural effusion: SDH 1.73, 95% CI 1.37–2.19, p < 0.001). Combined inclusion of PMA and pleural effusion improved NRI = 0.13 (p = 0.004) and IDI = 0.015 (p = 0.004) of overall mortality prediction compared to EuroSCORE II alone.

**Conclusions:**

Psoas muscle area (PMA) and pleural effusion were independently associated with cardiovascular mortality after TAVI, whereas no significant associations were observed with non-cardiovascular deaths. Combined inclusion of these parameters led to a modest but not clinically meaningful improvement in the EuroSCORE II–based prediction of mortality.

## 1 Introduction

Transcatheter aortic valve implantation (TAVI) is the treatment of choice for patients with severe aortic stenosis who are at high or prohibitive surgical risk [1]. Despite major procedural advances patient-related factors are critical in determining long-term benefit [2], [3]. Frailty is common among TAVI candidates [4], raising the question of whether these patients ultimately derive sustained benefit from the procedure. This underscores the need to identify markers of both cardiovascular and non-cardiovascular mortality that can guide patient selection, inform *peri*-procedural management, and highlight those requiring advanced follow-up strategies beyond the intervention itself.

The EuroSCORE II is a well-established surgical risk model originally developed to predict perioperative mortality, defined as in-hospital or 30-day death after cardiac surgery [5]. Although its primary purpose is perioperative risk assessment, there is also evidence that it correlates with mid- and long-term all-cause mortality after surgical and transcatheter aortic valve implantation [6], [7], [8]. While EuroSCORE II includes poor mobility as an indicator of severe functional limitation, it does not explicitly incorporate objective measures of sarcopenia or muscle mass, which may still contribute to long-term outcomes, particularly in elderly TAVI patients.

Sarcopenia, a core component of frailty, is traditionally assessed using physical performance tests [9], but these are often impractical in the procedural setting. A pragmatic alternative is measurement of the psoas muscle area (PMA), thereby offering a simple surrogate of sarcopenia without the need for additional testing. In parallel, pleural effusion, a radiographic marker of elevated filling pressures and advanced heart failure, is a frequent finding in TAVI candidates [10] and may further reflect limited cardiac reserve. Importantly, both PMA and pleural effusion can be evaluated from the same pre-procedural TAVI planning CT imaging, which can be quantified directly from the routine computed tomography (CT) scans obtained for TAVI planning [10], [11].

Given that sarcopenia may associate with non-cardiovascular death and thus limit the overall benefit of TAVI, we investigated whether CT-derived sarcopenia and pleural effusion are associated with cardiovascular or non-cardiovascular mortality. We further aimed to evaluate whether adding PMA and pleural effusion to the EuroSCORE II model could improve its ability to predict long-term all-cause mortality.

## 2 Methods

### 2.1 Study cohort

All consecutive patients undergoing transcatheter aortic valve implantation (TAVI) at Heart Hospital, Tampere University Hospital, Tampere, Finland, between 2008 and 2020 were included in this retrospective registry study. Patients with missing psoas muscle area measurements (n = 1) or pleural effusion assessments (n = 9) due to poor quality of CT imaging were excluded, resulting in a final study population of 1090 patients. Tays Heart Hospital is the sole provider of invasive cardiac care for approximately 500,000 residents in the Pirkanmaa region and serves as the only cardiothoracic surgery centre for a wider catchment area of around 1 million inhabitants.

### 2.2 Collection of clinical data and causes of death

Clinical background data, such as echocardiographic parameters, previous cardiac surgery or percutaneous coronary intervention and renal function, was acquired from the prospectively updated “KARDIO” registry maintained by cardiologists and thoracic surgeons, which is designed to collect relevant procedural and patient-related clinical data of patients undergoing invasive operations at the Heart Hospital, Tampere University Hospital. The data were further retrospectively updated by clinical information collected from the electronic hospital registry and by a full disclosure review of written patient records and charts.

We classified causes of death into three categories: (i) cardiovascular deaths, defined according to ICD-10 classification system (codes I00–I99); (ii) deaths due to non-cardiovascular diseases (e.g., cancers, infections); and (iii) deaths due to unnatural causes (e.g., accidents, poisonings). Information on causes of death was obtained from comprehensive nationwide data, Finnish mortality registry, which covers all deaths among Finnish citizens including those occurring abroad (Statistics Finland). [12] According to Finnish legislation, the attending physician must always issue a written death certificate, which includes clinical information on underlying diseases and the circumstances leading to death. In unclear cases, a medical autopsy is performed to verify the cause of death. Overall, approximately 21% of all deaths in Finland undergo autopsy. Most deaths due to unnatural causes are confirmed by medicolegal autopsy, unless the cause of death is unequivocally established by clinical findings or imaging.[13].

This study was conducted adhering to the ethical principles of the Helsinki Declaration and approved by the local institutional review board with permit number R20602. Due to the retrospective nature of this registry study, no formal patient consent was required or obtained.

## 3 Psoas muscle and pleural effusion measurements

Psoas measurements at the L3 level were primarily obtained from preoperative CT images. In cases where a preoperative scan was not available (<3% of patients), postoperative images were used instead, all acquired within 30 days after the operation. Median and interquartile range for the time between CT imaging and the TAVI procedure was 40 (IQR: 17–74) days. CT image measurements were performed in the contrast-enhanced arterial phase and axial slices thickness between 0.50–3.00 mm were used. Two different multidetector scanners were used: The General Electric LightSpeed 16-row scanner (GE Healthcare, Milwaukee, WI, USA) and the Philips Brilliance 64-row scanner (Philips Healthcare, Best, Netherlands) (∼80% with 100 kV and ∼20% with 120 kV). Both were in equal use and there was no selection between these scanners. CT images were reviewed using medical imaging workstations (Carestream Vue PACS viewer version 11.4.0.1253, Rochester, NY, USA). The psoas muscles were carefully outlined with free-hand tool by the authors of this study along the prominent muscle fascia after which the imaging workstation program automatically calculated the area of the outlined muscle in mm^2^ (Phillips Intellivue software (Philips Healthcare, Best, Netherlands). Left and right PMA measurements were combined, and the average PMA was calculated and used in all analyses. The thickness of the pleural fluid collection was measured in the posterior thoracic cavity from the chest wall to the lung surface at the point of maximal effusion. A threshold value of >10 mm was to define the presence of pleural effusion.

### 3.1 Statistical analysis

We used Cox regression model to assess the association of PMA and pleural effusion with overall mortality after TAVI. We then utilized a Fine-Gray subdistribution hazard models (SDH) to examine the association of PMA and pleural effusion with three predefined categories of mortality: cardiovascular deaths, deaths due to non-cardiovascular disease, and unnatural causes of death. To account for potential confounders, Cox and SDH models were adjusted for age, sex, BMI, and BSA. To further refine the analyses, the EuroSCORE II was added to the models.

We tested PMA and pleural effusion ability to improve EuroSCORE II prediction of overall mortality. A 3-year follow-up time was chosen to balance event numbers and follow-up completeness. Four models were compared: the baseline EuroSCORE II (M1), EuroScore II + PMA (M2), EuroSCORE II + pleural effusion (M3) and EuroSCORE II + PMA + pleural effusion (M4). Model discrimination was assessed using time-dependent AUCs (Harrell’s C-statistic) and integrated discrimination index (IDI). Re-classification was evaluated with continuous net-reclassification improvement (cNRI). Bootstrap resampling (500 iterations) was used in discrimination and re-classification estimates to obtain 95% confidence intervals. All statistical analysis were performed using Rstudio (version 1.3.109) with packages survIDINRI[14] and nricens[15]. A p-value <0.05 was considered statistically significant.

## 4 Results

The cohort included a total of 1090 patients with a median age of 81(IQR77.0–85.0) years, of whom 52.7% (574) were women. The median follow-up time for the entire population was 4.31(IQR 3.10–5.99) years and 54.1% (n = 590) of patients died during follow-up. The causes of death were distributed as follows: cardiovascular deaths 63.7% (n = 376), deaths due to non-cardiovascular diseases 30% (n = 177), and deaths due to unnatural causes 6.3%(n = 37). General demographics in the whole cohort and stratified by cause-of-death categories are presented in Table 1.

**Table 1:** General baseline characteristics in the entire TAVI population and stratified by cause-of-death categories.

Baseline characteric	All patients n = 1090	Alive at the end of follow-up 45.9% n = 500	Cardiovascular death 34.5% n = 376	Death due to non-cardiovascular disease 16.2% n = 177	Unnatural cause of death 3.4% n = 37
Age, years (median, IQR)	81(77.0–85.0)	80.0 (76–84)	83.0 (78.0–86.0)	82.0 (78.0–85.0)	84.0 (76.0–88.0)
Women, % (n)	52.7 (574)	53.4 (267)	54.0 (203)	44.1 (78)	70.3 (26)
L3 PMA mm^2^ mean (SD) *	724.3 (255.5)	746.3 (234.5)	700.4 (214.8)	734.0 (216.5)	624.3(209.5)
Transfemoral access, % (n)	88.5 (965)	43.9 (479)	31.7 (306)	15.75 (152)	2.9 (28)
BMI kg/m^2^, (mean, SD) kg/m^2^*	27.8 (5.9)	28.3 (6.8)	27.6 (5.1)	27.1(4.8)	25.9 (4.3)
BSA mm^2^ mean(SD)*	1.85(0.21)	1.87(0.21)	1.83(0.21)	1.86 (0.22)	1.75 (0.21)
Pleural effusion % (n) *	23.6 (257)	17.6 (88)	31.1 (117)	24.9 (44)	21.6 (8)
LVEF mean(SD)	54.9(12.8)	56.3 (12.0)	53.3(14.1)	53.9 (11.8)	55.9 (13.2)
Prior PCI % (n)	21.2(231)	19.8 (99)	21.8(82)	22.0 (39)	29.7 (11)
Prior heart surgery % (n)	17.3(189)	16.4 (82)	19.4(73)	15.3 (27)	18.9 (7)
eGFR ml/min/1.73 m^2^ mean(SD)	57.1(19.8)	61.4(18.9)	51.6(19.4)	57.6(20.5)	52.5(18.8)
Diabetes %(n)*	31.3(341)	29.8(149)	32.4(122)	33.9(60)	27.0(10)
COPD or asthma % (n)*	15.1(165)	13.2(66)	15.4(58)	20.3(36)	13.5(5)
Smoking or ex-smoker % (n)**	24.1(236)	25.7(114)	22.5(76)	25.3(41)	14.3(5)
Euroscore II* mean (SD)	7.16(7.43)	6.13(6.89)	8.71(8.37)	6.74(6.6)	7.43(4.91)
Median time to the end of follow-up or death (IQR)	4.31 (3.10–5.99)	5.08 (3.96–6.36)	3.20 (1.67–5.06)	3.47 (2.16–5.45)	3.59 (2.16–6.15)

Abbreviations: BMI = Body mass index; BSA = Body surface area; COPD = Chronic obstructive pulmonary disease; eGFR = Estimated glomerular filtration rate;IQR = Interquartile range; LVEF = Left ventricular ejection fraction; PCI = Percutaneous coronary intervention; PMA = Psoas muscle area; SD = Standard deviation.

* Data missing in <5%.

** Data missing in <15%.

## 5 Association of PMA and pleural effusion with all-cause and cause-sepcific mortality

Increase in psoas muscle area (PMA) was associated with a lower risk of all-cause mortality (HR/1SD 0.87, 95% CI 0.79–0.96, p = 0.005), whereas pleural effusion was associated with a higher risk (HR 1.50, 95% CI 1.25–1.81, p < 0.001) (Table 2). When PMA and pleural effusion were included in the same model, the hazard ratios remained unchanged (Table 2).

**Table 2:** The associations between psoas muscle area, pleural effusion and cause-specific deaths. The results are adjusted for age, sex, BMI and BSA.

**Overall Death; Cox model HR p-value 95%CI**
Psoas area (1SD). 0.87 0.005 0.79–0.96
Pleural effusion 1.50 < 0.001 1.25–1.81
Psoas area (1SD)* 0.87 0.005 0.79–0.96
Pleural effusion* 1.50 < 0.001 1.25–1.81
**Cardiovascular death; SDH model SDH**
Psoas area (1SD) 0.88 0.032 0.78–0.99
Pleural effusion 1.74 < 0.001 1.38–2.19
Psoas area (1SD)* 0.88 0.037 0.78–0.99
Pleural effusion* 1.73 < 0.001 1.37–2.19
**Death due to non-cardiovascular disease; SDH model**
Psoas area (1SD) 0.91 0.300 0.77–1.08
Pleural effusion 1.13 0.510 0.79–1.62
Psoas area (1SD)* 0.91 0.300 0.77–1.09
Pleural effusion* 1.13 0.510 0.79–1.62
**Unnatural cause of death; SDH model**
Psoas area (1SD) 0.72 0.150 0.45–1.13
Pleural effusion 1.08 0.840 0.51–2.27
Psoas area (1SD)* 0.71 0.150 0.45–1.12
Pleural effusion* 1.07 0.870 0.50–2.26

Abbreviations: CI = Confidence interval; HR = Hazard ratio according to Cox regression model; SD = Standard deviation; SDH = Subdistribution hazard according to Subdistribution hazard model.

* Psoas muscle area and pleural effusion analyzed in the same model.

In the subdistribution hazard models PMA was associated with lower cardiovascular mortality (SDH/1SD 0.88, 95% CI 0.78–0.99, p = 0.032), and pleural effusion with higher cardiovascular mortality (SDH 1.74, 95% CI 1.38–2.19, p < 0.001). When PMA and pleural effusion were included in the same model, the results were nearly identical (PMA SDH/1SD 0.88, 95% CI 0.78–0.99, p = 0.037; pleural effusion SDH 1.73, 95% CI 1.37–2.19, p < 0.001).

For deaths due to non-cardiovascular disease, neither PMA (SDH/1SD 0.91, 95% CI 0.77–1.08, p = 0.300) nor pleural effusion (SDH 1.13, 95% CI 0.79–1.62, p = 0.510) showed a statistically significant association. Similarly, for deaths due to unnatural causes, PMA (SDH/1SD 0.72, 95% CI 0.45–1.13, p = 0.150) and pleural effusion (SDH 1.08, 95% CI 0.51–2.27, p = 0.840) were not significantly associated with outcomes. (Table 2).

After further adjustment for EuroSCORE II, which already includes age and sex (Table 3), the findings remained largely consistent. An increase in PMA was associated with lower cardiovascular mortality (SDH/1SD 0.87, 95% CI 0.77–0.98, p = 0.025), but not with deaths due to non-cardiovascular disease (SDH/1SD 0.89, 95% CI 0.75–1.06, p = 0.180) or deaths due to unnatural causes (SDH/1SD 0.65, 95% CI 0.42–1.02, p = 0.059). Pleural effusion remained clearly associated with cardiovascular mortality (SDH 1.60, 95% CI 1.30–1.97, p < 0.001), without evidence of an association with deaths due to non-cardiovascular disease (SDH 1.21, 95% CI 0.84–1–74, p = 0.300) or unnatural causes (SDH 1.14, 95% CI 0.52–2.47, p = 0.750). When the analyses were restricted to patients undergoing transfemoral access TAVI (88.5%), the associations remained essentially unchanged (Supplementary Table S1).

**Table 3:** The associations between psoas muscle area, pleural effusion and cause-specific deaths. The results are adjusted for BMI, BSA and EuroSCORE II.

**Overall death; cox model BMI and BSA adjusted HR p-value 95%CI**
Psoas area(1SD) 0.85 0.001 0.78–0.94
Pleural effusion 1.46 < 0.001 1.21–1.77
EurSCOREII 1.07 0.069 0.99–1.15
**Cardiovascular death; SDH model BMI and BSA adjusted SDH**
Psoas area(1SD) 0.87 0.025 0.77–0.98
Pleural effusion 1.60 < 0.001 1.25–2.05
EuroSCOREII 1.13 0.009 1.03–1.23
**Death due to non-cardiovascular disease; SDH model BMI and BSA adjusted**
Psoas area(1SD) 0.89 0.180 0.75–1.06
Pleural effusion 1.21 0.300 0.84–1.74
EurosSCOREII 0.93 0.390 0.79–1.10
**Unnatural cause of death; SDH model BMI and BSA adjusted**
Psoas area(1SD) 0.65 0.059 0.42–1.02
Pleural effusion 1.14 0.750 0.52–2.47
EuroSCOREII 0.92 0.530 0.69–1.21

Abbreviations: CI = Confidence interval; HR = Hazard ratio; SD = Standard deviation; SDH = Subdistribution hazard model.

### 5.1 Discrimination and reclassification performance

The EuroSCORE II AUC for three-year survival prediction was 0.601. Adding PMA alone to the model did not improve discrimination (difference in AUC − 0.010, 95% CI − 0.029–0.049, p = 0.613). A minor but statistically significant improvement was observed in reclassification (IDI 0.006, 95% CI 0.000–0.014, p = 0.040), whereas the change in cNRI was not significant (0.083, 95% CI − 0.021 to 0.146, p = 0.084).

When pleural effusion was added to the EuroSCORE II model, discrimination remained unchanged (difference in AUC + 0.021, 95% CI − 0.048–0.005, p = 0.109). Small yet statistically significant improvements were observed in reclassification (IDI 0.010, 95% CI 0.002–0.019, p = 0.020; cNRI 0.140, 95% CI 0.072–0.202, p = 0.008).

Combining both PMA and pleural effusion with EuroSCORE II did not result in a statistically significant difference in discrimination (difference in AUC + 0.015, 95% CI − 0.057 to 0.028, p = 0.492). Significant improvements were observed in reclassification (IDI 0.015, 95% CI 0.004–0.029, p = 0.004; cNRI 0.129, 95% CI 0.060–0.212, p < 0.001) (Table 4).

**Table 4:** Discrimination and reclassification performance of PMA and pleural effusion above EuroSCORE II alone in three (3) years follow-up.

Model	AUC (3 y, 95% CI)	ΔAUC (95% CI)	p-value	cNRI (95% CI)	p-value	IDI (95% CI)	p-value
M1	0.601 (0.563–0.640)	NA	NA	NA	NA	NA	NA
M2	0.591 (0.552–0.631)	−0.010 (−0.029– 0.049)	0.613	0.083 (−0.021– 0.146)	0.084	0.006 (0.000–0.014)	0.040
M3	0.623 (0.585–0.661)	+0.021 (−0.048–0.005)	0.109	0.140 (0.072– 0.202)	0.008	0.010 (0.002 –0.019)	0.020
M4	0.616 (0.578–0.656)	+0.015 (−0.057–0.028)	0.492	0.129 (0.060–0.212)	<0.001	0.015 (0.004– 0.029)	0.004

Abbreviations: AUC = area under the receiver operating characteristic curve; IDI = integrated discrimination improvement; cNRI = continuous net reclassification improvement; CI = confidence interval; NA = not applicable; M1 = EuroSCORE II, M2 = EuroSCORE II + psoas muscle area (PMA); M3 = EuroSCORE II + pleural effusion; M4 = EuroSCORE II + PMA + pleural effusion.

## 6 Discussion

By examining 1090 patients undergoing TAVI with a median follow-up of 4.3 years, we made four key findings. First, CT-derived sarcopenia, assessed by psoas muscle area (PMA), was associated with cardiovascular mortality but showed no association with competing non-cardiovascular death. Second, pre-procedural CT-detected pleural effusion was strongly associated with cardiovascular mortality but not with non-cardiovascular death. Third, both PMA and pleural effusion were independently associated with mortality beyond the widely used EuroSCORE II. Fourth, when both PMA and pleural effusion were added to the EuroSCORE II model, the predicted risk moved in the correct direction for approximately 13% of patients indicating that the predictions became more accurate, although the overall improvement was small.

Despite advances in technique and patient selection, mortality among TAVI recipients remains high—45.7% at 4.3 years in the present cohort. During long-term follow-up after the procedure, patients often experience progressive worsening of frailty, increasing comorbidity burden, and recurrent hospitalizations, reflecting the overall decline in health status despite procedural success [4].

Although EuroSCORE II is widely used for prognostication, it only partially captures patient frailty through the poor mobility variable and does not directly account for sarcopenia or quantitative measures of muscle mass. Together with the high incidence of non-cardiovascular mortality as a competing risk, this may attenuate the overall benefit of TAVI in vulnerable patients. In addition, EuroSCORE II does not include imaging-based or biomarker measures reflecting congestion or subclinical heart failure, which are important cardiovascular determinants of outcome. In the present study, pre-procedural CT-detected pleural effusion—a hallmark of cardiac congestion—was associated with higher cardiovascular mortality without a corresponding increase in competing non-cardiovascular deaths, highlighting the need for closer surveillance and tailored heart failure management in these patients. Although episodes of decompensation and congestion in severe aortic stenosis are often attributed to the valvular lesion itself, and TAVI reduces the rate of heart failure hospitalizations compared with the pre-procedural period [16], this reduction is modest [17]. This underscores the importance of preventive strategies beyond valve replacement. Recent evidence further supports this view: treatment with dapagliflozin was shown to reduce the composite of all-cause mortality and heart failure hospitalization in patients with severe aortic stenosis undergoing TAVI, an effect largely driven by the reduction in heart failure admissions [18]. Together, these findings suggest that pleural effusion is a clinically meaningful imaging biomarker of cardiovascular vulnerability in the TAVI population.

CT-derived sarcopenia, assessed by psoas muscle area (PMA), was independently associated with cardiovascular mortality but not with non-cardiovascular causes. Accumulating evidence suggests that larger PMA is linked to lower mortality in TAVI patients [11], [19]. Importantly, in our study this association was evident specifically for cardiovascular mortality, with each one standard deviation increase in PMA associated with a 12% reduction in risk, even after adjustment for key clinical covariates and EuroSCORE II. This finding suggests that lower PMA reflects diminished physiological reserve and increased frailty, which are particularly relevant in older patients undergoing major cardiovascular procedures. PMA showed no association with non-cardiovascular mortality, which may indicate that sarcopenia primarily influences outcomes through mechanisms related to cardiovascular vulnerability rather than through comorbidities outside the cardiovascular system. Our findings extend previous observations on the prognostic role of psoas muscle area (PMA) in cardiovascular patients undergoing invasive procedures. In line with earlier reports [19], [20], [21], [22], [23], [24], [25], [26], [27], [28], [29], [30], [31], we observed that a larger PMA was associated with lower overall mortality, and this association appeared linear across the normal deviation in PMA values [32]. PMA may represent a potentially modifiable risk factor. Targeted resistance training has been shown to improve muscle mass, strength, and physical performance in older adults, and recent evidence suggests it may also be associated with reduced mortality, although causality remains uncertain [33], [34]. These findings highlight the potential clinical relevance of sarcopenia-focused interventions in patients undergoing TAVI.

Beyond muscle-related frailty markers, we also found that pleural effusion—a common manifestation of advanced heart failure, which develops in more than 30% of patients with severe aortic stenosis [35], [36], [37] was present in a substantial proportion of our cohort. While only few studies have examined the prognostic impact of preoperative pleural effusion in TAVI [10], [38] and none have assessed its association with specific causes of death, our results provide new insights by directly comparing the relative contribution of PMA and pleural effusion to cardiovascular versus non-cardiovascular mortality. Both low PMA and pleural effusion were independently associated with mortality beyond EuroSCORE II. This extends prior observations on the prognostic role of sarcopenia and congestion markers in structural heart disease.

Although both low psoas muscle area and pleural effusion were independently associated with mortality, their inclusion in the EuroSCORE II–based prediction model did not meaningfully improve discrimination. While risk reclassification improved by about 13%, the average improvement of 1.5 percentage units (IDI = 0.015) can be considered modest. This indicates that while these imaging-derived measures capture relevant aspects of frailty and physiological vulnerability, the prognostic information they provide largely overlaps with factors already represented within the EuroSCORE II components, such as age and comorbidities. EuroSCORE II does not explicitly incorporate markers of sarcopenia or congestion, yet its variables indirectly reflect these conditions. As a result, adding PMA and pleural effusion offered limited incremental value despite their strong independent associations with outcomes. This distinction underscores a common phenomenon in risk modeling: a variable may be biologically and statistically linked to mortality but still fail to enhance overall model performance when the baseline model already encapsulates much of the same risk information. Together, these findings suggest that while PMA and pleural effusion move the EuroSCORE II model in the right direction, they do not improve discrimination in a clinically meaningful way, although the presence of pleural effusion should nonetheless prompt initiation of effective heart failure therapy, which has been shown to improve prognosis [18].

From a clinical perspective, both low PMA and pleural effusion may help identify TAVI patients who warrant closer evaluation or follow-up. Low PMA reflects reduced physiological reserve and low habitual activity, and such patients may potentially benefit from structured rehabilitation after TAVI. Although resistance training improves muscle mass and physical performance in older adults, its effect on mortality in the TAVI population remains unknown; therefore, PMA should be viewed primarily as a marker of vulnerability rather than a modifiable therapeutic target. Very low PMA in a seemingly asymptomatic patient may also indicate low activity levels that mask exertional symptoms, supporting consideration of earlier intervention in selected cases. In contrast, pleural effusion reflects overt congestion and should prompt timely optimization of heart failure therapy or avoidance of delays in TAVI, given that effective decongestion has been associated with improved outcomes in patients with severe aortic stenosis. Overall, these findings suggest that while PMA and pleural effusion do not meaningfully enhance formal risk prediction, they provide clinically useful context for patient assessment, procedural timing, and post-TAVI management. Finally, whether targeted resistance training before or after TAVI can improve prognosis remains unknown and warrants further study. This is further supported by observations from another cardiovascular population—abdominal aortic aneurysm patients undergoing endovascular aortic repair (EVAR)—in whom longitudinal declines in PMA predicted worse survival [39].

A notable strength of this study is the relatively large national cohort of 1090 TAVI patients with complete follow-up and no loss to follow-up. The accuracy of cause-of-death classification is supported by the nationwide Finnish mortality registry, in which all deaths are physician-certified and reviewed by medico-legal experts. In unclear cases, autopsies are performed according to legal requirements, and approximately one-fifth of all deaths in Finland are subject to autopsy [13]. Because of the retrospective design, the associations observed in this study cannot establish causality. Despite adjustment for EuroSCORE II, several potentially important confounders—such as nutritional status, inflammatory burden, habitual physical activity, and detailed frailty metrics—were not available and may influence both PMA, pleural effusion, and mortality. It should also be noted that EuroSCORE II was originally developed for short-term operative risk assessment, and its applicability for long-term prognostication may therefore be limited, which could partly explain the modest incremental improvements observed when PMA and pleural effusion were added. Finally, this single-centre study included exclusively elderly Finnish patients, which may limit generalizability to more diverse populations.

## 7 Conclusions

Psoas muscle area and pleural effusion were independently and complementarily associated with cardiovascular mortality after TAVI, but not with non-cardiovascular deaths. Although they did not improve the long-term predictive performance of EuroSCORE II, these imaging markers may still offer practical value in identifying frail TAVI patients who require closer follow-up.

## Ethical approval

This study was conducted adhering to the ethical principles of the Helsinki Declaration and approved by the local institutional review board with permit number R20602. Due to the retrospective nature of this registry study, no formal patient consent was required or obtained.

## CRediT authorship contribution statement

**Otto Järvinen:** Writing – review & editing, Writing – original draft, Validation, Methodology, Investigation, Formal analysis, Conceptualization. **Jani Rankinen:** Writing – review & editing, Writing – original draft. **Jussi Hernesniemi:** Writing – review & editing, Supervision, Resources, Project administration, Methodology, Investigation, Funding acquisition, Data curation, Conceptualization. **Marko Virtanen:** Writing – review & editing, Investigation. **Pasi Maaranen:** Investigation. **Markku Eskola:** Writing – review & editing. **Niku Oksala:** Writing – review & editing. **Juho Tynkkynen:** Writing – review & editing, Supervision, Methodology, Formal analysis.

## Funding

This study was supported by competitive research funding from the Pirkanmaa Wellbeing Area, the Tays Support Foundation, the Finnish Foundation for Cardiovascular Research, and the European Commission (Horizon Europe, CVD-LINK).

## Declaration of competing interest

The authors declare that they have no known competing financial interests or personal relationships that could have appeared to influence the work reported in this paper.
