# Supplementary material for: Prognostic value of psoas muscle area and pleural effusion in patients undergoing TAVI
Source: Int J Cardiol Heart Vasc. 2026 Jan 18;62:101871. doi: 10.1016/j.ijcha.2026.101871 (PMC12856589; doi:10.1016/j.ijcha.2026.101871)
Supplement: Supplementary Data 1 [file mmc1.docx]

Supplementary Table S1. The associations between psoas muscle area, pleural effusion and cause-specific deaths in patients undergoing transfemoral access TAVI, adjusted for BMI, BSA and EuroSCORE II.

| **Overall death; cox model BMI and BSA adjusted** **HR p-value 95%CI**  Psoas area(1SD) 0.82 <0.001 0.74–0.91  Pleural effusion 1.41 0.002 1.14­–1.76  EuroSCOREII 1.01 0.095 1.00­–1.02  **Cardiovascular death; SDH model BMI and BSA adjuste SDH**  Psoas area(1SD) 0.87 0.048 0.76–1.00  Pleural effusion 1.38 0.034 1.03–1.85  EuroSCOREII 1.02 0.008 1.01–1.04  **Death due to non-cardiovascular disease ; SDH model BMI and BSA adjusted**  Psoas area(1SD) 0.93 0.37 0.78–1-10  Pleural effusion 1.14 0.51 0.78–1.56  EuroSCOREII 0.98 0.09 0.95–1.00  **Unnatural cause of death; SDH model BMI and BSA adjusted**    Psoas area(1SD) 0.64 0.091 0.38–1.07  Pleural effusion 1.14 0.75 0.50–2.59  EuroSCOREII 0.97 0.25 0.92–1.02 |
| --- |

**Abbreviations:**
CI = Confidence interval; HR = Hazard ratio; SD = Standard deviation; SDH = Subdistribution hazard model.
